# The small bowel microbiome changes significantly with age and aspects of the ageing process

**DOI:** 10.15698/mic2022.01.768

**Published:** 2021-12-27

**Authors:** Gabriela Leite, Mark Pimentel, Gillian M. Barlow, Ruchi Mathur

**Affiliations:** 1 Medically Associated Science and Technology (MAST) Program, Cedars-Sinai, Los Angeles, CA, USA.; 2 Karsh Division of Gastroenterology and Hepatology, Department of Medicine, Cedars-Sinai, Los Angeles, CA, USA.; 3 Division of Endocrinology, Diabetes, and Metabolism, Department of Medicine, Cedars-Sinai, Los Angeles, CA, USA.

**Keywords:** age, aging, small intestinal microbiome, medication use, concomitant diseases, proteobacteria, coliforms

## Abstract

Gut microbiome changes have been associated with human ageing and implicated in age-related diseases including Alzheimer's disease and Parkinson's disease. However, studies to date have used stool samples, which do not represent the entire gut. Although more challenging to access, the small intestine plays critical roles in host metabolism and immune function. In this paper (Leite *et al.* (2021), Cell Reports, doi: 10.1016/j.celrep.2021.109765), we demonstrate significant differences in the small intestinal microbiome in older subjects, using duodenal aspirates from 251 subjects aged 18-80 years. Differences included significantly decreased microbial diversity in older subjects, driven by increased relative abundance of phylum Proteobacteria, particularly family Enterobacteriaceae and coliform genera *Escherichia* and *Klebsiella.* Moreover, while this decreased diversity was associated with the ‘ageing process' (comprising chronologic age, number of medications, and number of concomitant diseases), changes in certain taxa were found to be associated with number of medications alone (*Klebsiella*), number of diseases alone (*Clostridium, Bilophila*), or chronologic age alone (*Escherichia*, *Lactobacillus, Enterococcus*). Lastly, many taxa associated with increasing chronologic age were anaerobes. These changes may contribute to changes in human health that occur during the ageing process.

## EXPLORING THE INFLUENCE OF AGE AND AGEING ON THE SMALL INTESTINAL MICROBIOME

The human gut microbiome, comprising bacteria, archaea, fungi, parasites, and viruses, has numerous, significant impacts on the physiology of the human host throughout its lifespan, including roles in nutrient absorption and metabolism, immune function, and even brain function and behavior. Following rapid colonization at birth, the gut microbiome undergoes dynamic changes during early childhood before settling into a relatively stable pattern that was thought to persist throughout adulthood, unless impacted by significant changes in diet, medications, or disease. However, recent studies have demonstrated that the gut microbiome changes with increasing age and that the diversity of the gut microbiome may influence longevity and healthy ageing. A caveat is that these, like the majority of gut microbiome studies, relied on stool samples. Although stool is easier to procure and analyze, the small intestine is central to metabolism and the maintenance of homeostasis, and its microbial populations are significantly different from those in stool. Therefore, to explore the effects of ageing specifically on the small intestinal microbiome, we procured a total of 251 duodenal aspirates from subjects aged 18 to 80 years which had been collected as part of the REIMAGINE (Revealing the Entire Intestinal Microbiota and its Associations with the Genetic, Immunologic, and Neuroendocrine Ecosystem) study. REIMAGINE was created to explore the relationships between small intestinal microbial populations and human health and disease, using small intestinal aspirate samples obtained from subjects undergoing standard of care upper endoscopy without colon preparation. Samples are collected using novel, validated techniques designed to prevent contamination from saliva and the stomach, and maximize recovery of DNA from small intestinal microbiota. Subjects complete a comprehensive medical history questionnaire, and also provide blood, stool, saliva, and other samples, allowing us to correlate changes in small intestinal microbial populations with levels of serum biomarkers as well as microbial populations in other parts of the gastrointestinal tract. Cognizant of the fact that numbers of underlying conditions, and hence medication use, can increase with increasing age, and are also confounders in the study of gut microbes, we used the term ‘ageing process' to include chronological age, number of concomitant diseases, and number of medications, and sought to distinguish their separate and collective effects on the duodenal microbiome.

## ASPECTS OF THE AGEING PROCESS AFFECT METABOLIC AND INFLAMMATORY MARKERS

Comparing four groups of subjects (18-35 years, 36-50 years, 51-65 years, and 66-80 years, total N=251), increasing fasting blood glucose levels were found to be associated both with increasing chronologic age and with increasing number of medications. Consistent with our findings, age-related changes in glucose metabolism have previously been associated with increased risks for hyperglycemia and cardiovascular disease. Levels of the pro-inflammatory cytokine TNFα were found to be influenced by the ageing process (i.e. they increased with increasing chronologic age but also with number of medications and diseases), whereas IL8 levels were influenced by chronologic age alone. Consistent with these findings, several age-related diseases have been suggested to result, at least in part, from changes in the gut microbiome and resulting increases in inflammatory responses to microbial polysaccharides, including Alzheimer's disease and Parkinson's disease.

## DUODENAL MICROBIAL DIVERSITY AFFECTED BY THE AGEING PROCESS AND BY COLIFORM NUMBERS

**Figure 1:**
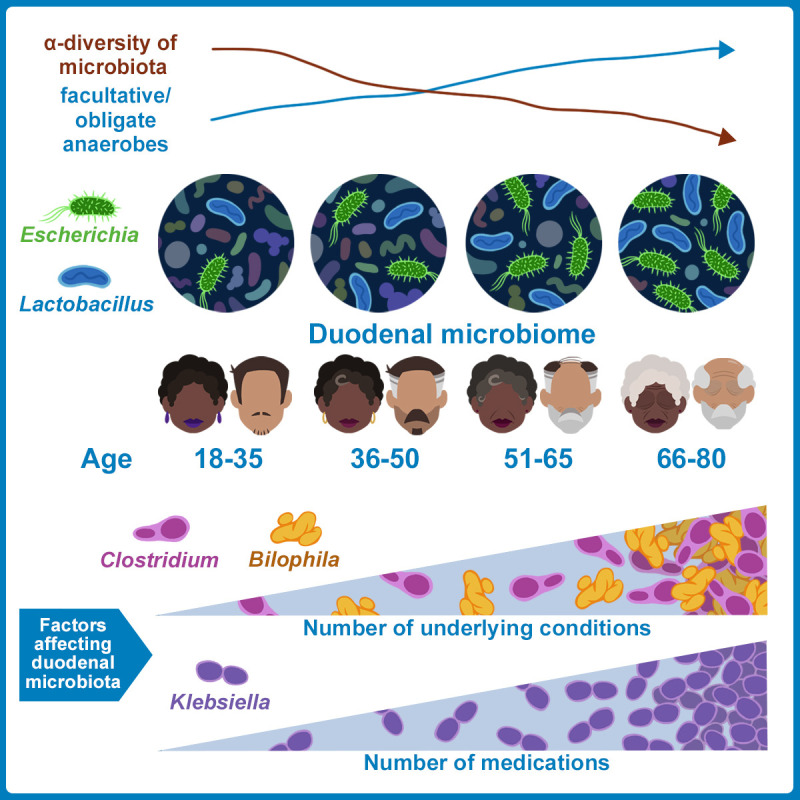
FIGURE 1: Effects of age and aspects of the ageing process on the small bowel microbiome. Our findings indicate that the duodenal microbiome changes progressively and significantly in older subjects. This includes decreased microbial diversity, increased prevalence of phylum Proteobacteria and anaerobic microbes, increased coliform levels, and decreased prevalence of phylum Bacteroidetes. Changes in specific genera correlated with chronologic age alone (*Bacteroides, Lactobacillus, Escherichia*), number of medications (*Klebsiella*), or number of underlying conditions/concomitant diseases (*Clostridium*).

The diversity of duodenal microbial populations was found to be significantly decreased in older subjects. This is consistent with previous studies showing decreased stool microbiome diversity with age. However, multivariate analyses revealed that these decreases were driven not only by chronologic age, but also by the number of diseases and number of medications used (by the ageing process rather than by chronologic age alone) (see **Figure 1**). In addition, microbial culture analyses revealed a striking association between the decreased duodenal microbial diversity in older subjects and increases in the numbers of live coliform bacteria, particularly the genera *Escherichia* and *Klebsiella* (phylum Proteobacteria). This was significant as increases in coliform bacteria, which normally reside in the colon and are found in feces, have also been demonstrated in microbiome-associated diseases of the small bowel (e.g. small intestinal bacterial overgrowth and inflammatory bowel disease) and are also associated with increased inflammatory responses.

## THE PREVALENCE OF PHYLUM BACERTIOIDES DECREASES, AND PROTEOBACTERIA INCREASES IN OLDER SUBJECTS

The core duodenal microbiome (the most widespread microbial components of the microbiome that are found across all subjects) changed progressively in older subjects. Although phylum Firmicutes was the most prevalent across all ages, the prevalence of phylum Bacteroidetes decreased in older subjects, going from the 3^rd^ most prevalent in Group 1 (18-35 years) to the 5^th^ most prevalent in Groups 2-4. This is consistent with previous findings from the stool microbiome and is also consistent with findings from our other small intestinal microbiome studies, which have continually identified strong correlations between duodenal microbial diversity and the abundance of phylum Bacteroidetes, particularly family Prevotellaceae and genus *Prevotella*.

In contrast, the prevalence of phylum Proteobacteria increased with increasing age, going from the 5^th^ most prevalent in Group 1 to the 2^nd^ most prevalent in Groups 2-4. This increased prevalence of Proteobacteria resulted in significantly altered correlations between Proteobacteria and other phyla, Firmicutes and TM7, suggesting a disruption of the core duodenal microbiome. Further analysis revealed that the increased prevalence of Proteobacteria in older subjects was driven primarily by the family Enterobacteriaceae, and specifically the coliform genera *Escherichia* and *Klebsiella*, which was consistent with our microbial culture findings.

Interestingly, increased prevalence of Enterobacteriaceae correlated directly with decreased duodenal microbial diversity, and Enterobacteriaceae also had the greatest number of negative associations with other duodenal families, further suggesting that the increased relative abundance (RA) of Enterobacteriaceae in older subjects results in a disruption of the core duodenal microbiome and decreased duodenal microbial diversity. In addition, the increased RA of the genera *Escherichia* and *Klebsiella* correlated with alterations in predicted duodenal microbial metabolic pathways, including a predicted upregulation of the antioxidant ubiquinone biosynthesis pathway. This prediction is interesting as ubiquinone plays an essential role in respiration in *E. coli,* limiting oxidative stress, and is upregulated in anoxic environments.

## CHANGES IN SOME DUODENAL TAXA ARE DRIVEN BY CHRONOLOGIC AGE ALONE, WHEREAS OTHERS ARE DRIVEN BY NUMBER OF DISEASES OR MEDICATION USE

In addition to the analyses of the common core duodenal microbiome across all age groups described above, the duodenal microbial profiles in the youngest (18-35 years) and oldest (66-80 years) were directly compared. The RA of families Enterobacteriaceae (phylum Proteobacteria) and Lactobacillaceae (phylum Firmicutes) were significantly increased in the oldest as compared to the youngest adults, and further analyses showed that this was driven by significantly increased relevant abundances of three genera, *Klebsiella* and *Escherichia* (family Enterobacteriaceae) and *Lactobacillus* (family Lactobacillaceae). This is significant as we have consistently found that these three genera act as ‘disruptors' of the small intestinal microbiome and are associated with loss of microbial diversity. Significantly, multivariate analysis revealed that the increase in RA of *Klebsiella* in older subjects was in fact driven solely by the number of medications used, regardless of age or number of diseases present, whereas *Escherichia* and *Lactobacillus* were driven by increases in chronological age alone and were not associated with medication use or number of diseases (see **Figure 1**). In addition to these three major genera, the RA of other duodenal taxa were also driven by components of the ageing process – for example, the increased RA of genus *Enterococcus* in older subjects was associated with chronological age alone, whereas the genera *Clostridium* (phylum Firmicutes) and *Bilophila* (phylum Proteobacteria) were driven by number of diseases only.

## INCREASED PREVALENCE OF FACULTATIVE AND OBLIGATE ANAEROBES IN THE DUODENAL MICROBIOME IN OLDER SUBJECTS IS ASSOCIATED WITH CHRONOLOGICAL AGE ALONE

In total, our multivariate analyses identified seven genera whose RA were associated with chronologic age alone and were not influenced by medication use or number of diseases. Of these, four were found to be facultative, obligate, or aerotolerant anaerobes, *Escherichia, Lactobacillus, Actinobacillus* (phylum Proteobacteria)*,* and *Bacteroides* (phylum Bacteroidetes), and exhibited increased RA in older subjects (see **Figure 1**). In contrast, the RA of an unknown genus from the strictly aerobic family Xanthomonadaceae (phylum Proteobacteria) was consistently decreased in the duodenal microbiome of older subjects. This increased RA of anaerobes is consistent with the predicted upregulation of the ubiquinone biosynthesis pathway in older subjects, which occurs in anoxic environments.

In conclusion, this first examination of the effects of age and the ageing process on the small intestinal microbiome demonstrates that the duodenal microbiome changes with increasing age, with significant decreases in duodenal microbial diversity due to increased prevalence of phylum Proteobacteria, particularly coliforms and anaerobic taxa. Given the key roles of small intestinal microbes in nutrient absorption and host metabolism, these changes may be clinically relevant for human health during the ageing process.

